# Radiation Absorbed Dose to the Basal Ganglia from Dopamine Transporter Radioligand ^**18**^F-FPCIT

**DOI:** 10.1155/2014/498072

**Published:** 2014-06-30

**Authors:** William Robeson, Vijay Dhawan, Yilong Ma, David Bjelke, Claude Margouleff, Thomas Chaly, David Eidelberg

**Affiliations:** ^1^Radiation Safety Office, The Feinstein Institute for Medical Research, 350 Community Drive, Manhasset, NY 11030, USA; ^2^Center for Neurosciences, The Feinstein Institute for Medical Research, 350 Community Drive, Manhasset, NY 11030, USA

## Abstract

Our previous dosimetry studies have demonstrated that for dopaminergic radiotracers, ^18^F-FDOPA and ^18^F-FPCIT, the urinary bladder is the critical organ. As these tracers accumulate in the basal ganglia (BG) with high affinity and long residence times, radiation dose to the BG may become significant, especially in normal control subjects. We have performed dynamic PET measurements using ^18^F-FPCIT in 16 normal adult subjects to determine if in fact the BG, although not a whole organ, but a well-defined substructure, receives the highest dose. Regions of interest were drawn over left and right BG structures. Resultant time-activity curves were generated and used to determine residence times for dosimetry calculations. *S*-factors were computed using the MIRDOSE3 nodule model for each caudate and putamen. For ^18^F-FPCIT, BG dose ranged from 0.029 to 0.069 mGy/MBq. In half of all subjects, BG dose exceeded 85% of the published critical organ (bladder) dose, and in three of those, the BG dose exceeded that for the bladder. The BG can become the dose-limiting organ in studies using dopamine transporter ligands. For some normal subjects studied with F-18 or long half-life radionuclide, the BG may exceed bladder dose and become the critical structure.

## 1. Introduction

Neurology and psychiatry PET research has focused on compounds that localize in the basal ganglia (BG) and trace different functions of the dopaminergic pathway. We have studied three of these compounds: ^18^F-FDOPA, ^11^C-raclopride, and ^18^F-FPCIT. All of them localize in the BG with varying affinity. Published dosimetry has demonstrated that whole brain is not limiting for these compounds, but the BG substructures may be [1–3].

Our previous paper in* J Nucl Med* [2] was a whole body PET study designed to estimate dosimetry for all organs especially the bladder (bladder was found to be the critical organ in our previous dosimetry studies for ^18^F-FDOPA [1]). This study demonstrated that the critical organ was indeed the bladder with estimated dose of 0.217 rads/mCi and the maximum allowable injected dose was 23 mCi. Even though the current study was performed to determine the kinetics of ^18^F-FPCIT in the basal ganglia of normal subjects, we realized that this data could also be used for estimating radiation dose to these structures.

This particular study of ^18^F-FPCIT was undertaken to determine if, in subjects for which dynamic PET data was available, the BG structures were dose-limiting. With increasing clinical use of longer half-life labeled dopamine transporter radioligand, ^123^I-FPCIT (DatScan), this issue has become even more relevant. Previous dosimetry work has been focused on anatomically well-defined organs while the current work takes into account both anatomy and significant functional differences within the organs themselves. Uptake of dopaminergic radiotracers is a case in point where uptake, as well as the residence time, is far greater in BG (a well-defined substructure within the brain) compared to the rest of the brain. This observation leads us to believe that the radiation burden to BG will be higher than the rest of the brain. The same logic can, in the future, be used to treat renal cortex as distinct from the kidneys if new radiotracers were to specifically target these locations. The principle is similar to that of labeled monoclonal antibody therapy where the radionuclide binds to the tumor surface sites and delivers the required therapeutic radiation dose. The tumor can be small or large, homogeneously or heterogeneously distributed. This is in contrast to the BG, which is anatomically well-delineated and homogeneous at least as far as dopaminergic radiotracer uptake is concerned in normal subjects. The uptake of dopaminergic tracers in normal control subjects provides the worst-case scenario for dosimetry. All these studies were conducted under Radioactive Drug Research Committee approved protocols where the dose limits are organ-based. Finally, another issue that needs to be addressed is the question of radiosensitivity of brain compared to other organs. We do not yet have any data to suggest that BG is more radiosensitive than the rest of the brain (which itself is not very radiosensitive compared to reproductive organs) [4]. However, there is enough evidence to suggest that BG is more sensitive to hypoxia and has larger mitochondrial load and dopamine levels than the rest of the cortical structures. These differences can potentially lend BG to be relatively more sensitive to radiation than the rest of the brain [5].

## 2. Methods and Materials

Dynamic PET scans of the brain were acquired in 16 normal adult subjects. Data were acquired in 3D mode on a GE Advance tomograph (General Electric, Milwaukee, WI). Scanning protocol included 21 frames: 5 × 1 min, 5 × 2 min, 5 × 5 min, and 6 × 10 min. Ethical permission for the procedures was obtained from the Institutional Review Board of North Shore University Hospital. Written consent was obtained from each participant following a detailed explanation of the procedures. Thirty-five slices parallel to the orbital-meatus were collected over an axial field of view of 15 cm so that the entire brain was covered. Emission data were corrected for attenuation using a rotating Ge-68 source. Image reconstruction was performed using filtered backprojection with a cutoff resolution of 8 mm. All brain slices that included the BG were added to form a composite image. Regions of interest (ROI) were drawn over the left caudate, left putamen, right caudate, and right putamen, and time-activity curves were generated over the duration of the scan. No MRI scans were available for these normal subjects. Also, it was assumed that for dosimetry purposes, the ROI drawn on PET scans was sufficient without an MRI coregistration. Activity concentrations (kBq/cc) were computed using a calibration scan of a cylindrical phantom of known activity concentration. Time-activity curves for the four BG regions were fit to a nonlinear regression model of exponential uptake and clearance and analytical integration was employed to estimate the area under the curve (AUC). Units for AUC are (kBq/cc)∗(min). For each subject and each region, normalized AUC/MBq was calculated and then multiplied by mass (grams) and the *S*-value (mGy/kBq∗min) as follows:

(1) $$\begin{array}{c} \text{Dose}\;\left(\text{mGy}/\text{MBq}\right)=\left(\frac{\text{AUC}}{\text{MBq}}\right)*\text{mass}*S\text{-value}. \end{array}$$

We did not have individual MRI images available for estimating BG volumes. Therefore, BG structures were assumed to be spherical regions of mass 3.7 and 4.4 grams for caudate nuclei and putamen, respectively, in each brain hemisphere; these were derived from average masses of BG structures from seven published MRI datasets [6–12]. We could not use age dependent masses because the published data demonstrate no clear relationship between age and BG volume [6–12]. *S*-factors for dosimetry calculations were determined from interpolation based upon data from the MIRDOSE 3 nodule module [13]. Doses were calculated for the BG from activity in the BG substructures which were then averaged and compared with published critical organ values. Given that ^18^F-FPCIT uptake decreases with age, we also investigated the relationship between dose to the BG and age [14].

## 3. Results

**Figure 1:**
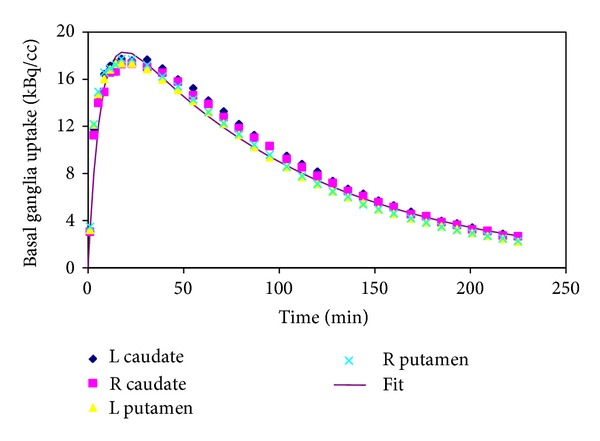
Dynamic ^18^F-FPCIT PET regions of interest data from basal ganglia fitted to extract residence times. Data from four regions of interest is presented (caudate and putamen on left and right hemispheres). A multiexponential curve fit to the right putamen data is also shown. Analytical area under the curve was obtained from this fitted curve and dose estimated using (1) (see text).

Figure 1 demonstrates an example of the fitting procedure used to calculate BG doses. Data from all four substructures from one subject are shown but only the right putamen data has been fitted to demonstrate a typical example of curve fit.

**Figure 2:**
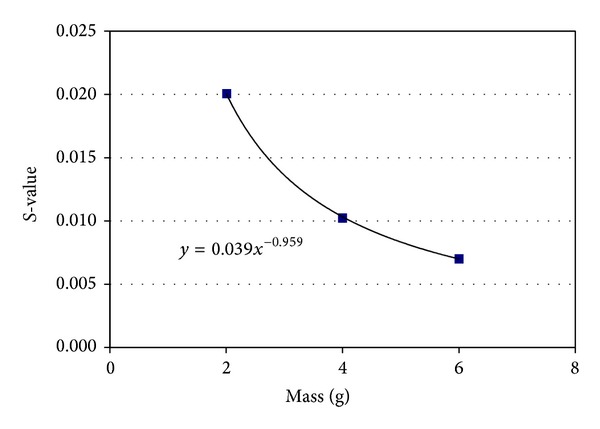
*S*-values as a function of mass. *S*-values were calculated for different masses. A range from 2 to 6 grams covers the expected caudate and putamen masses [6–12]. A power relationship provides a good fit to the data.

Figure 2 presents *S*-values as a function of mass. A mass from 2 to 6 grams covers the range expected for caudate and putamen substructures of the BG [6–12]. A power relationship (*S* = 0.039∗mass^−0.959^) provides a good fit to the data.

Table 1 presents the results for the BG doses for the 16 subjects studied. BG doses are compared to published values for critical organ (bladder) dose established in the literature. The average dose to the BG was approximately 81% of the bladder dose (bladder dose was obtained as 0.059 mGy/MBq from reference [2]). In half of the subjects (*n* = 8) the BG dose was close to the dose to the urinary bladder (>85%). In three out of 16 subjects the BG dose exceeded the dose to the bladder (Table 1,* bold italics*). Therefore, in three subjects, the BG became the critical “organ” (not traditionally defined as an organ but a substructure in the brain).

**Figure 3:**
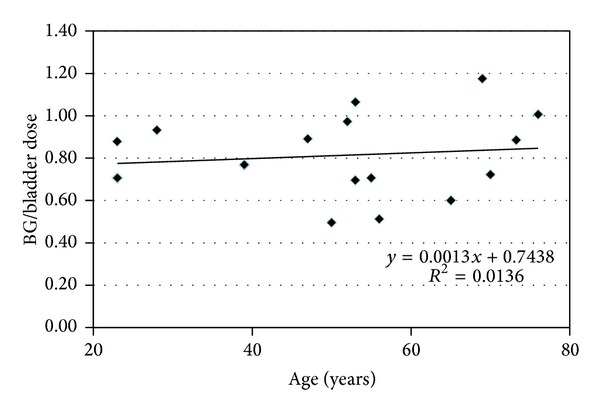
Ratio of basal ganglia and bladder dose as a function of age. In three out of 16 subjects, basal ganglia (BG) dose exceeded that of the critical organ (urinary bladder) for ^18^F-FPCIT. In half of the subjects (*n* = 8), the BG dose exceeded 85% of the bladder dose. No aging effect on the BG dose was observed (*R* ^2^ = 0.0136, *P* = 0.67).

Figure 3 presents graphically the data from Table 1 demonstrating that in three subjects the dose to the BG exceeded that of the urinary bladder (critical organ for ^18^F-FPCIT) and the regression line between age and BG dose. No relationship between age and BG dose could be demonstrated (*R* ^2^ = 0.0136, *P* = 0.67). BG dose based upon individually derived measures of BG volume could provide a better age versus dose relationship.

## 4. Discussion

PET studies utilizing radiopharmaceuticals that localize in the BG have become very useful in patients with movement disorders. Radiation dosimetry using the MIRD techniques has traditionally evaluated whole organ doses. Using standard dosimetry in humans with F-18 PET compounds, the urinary bladder is the critical organ. Neuroleptic compounds localize in the BG, and although the whole brain might not be critical, BG may be. We have used the MIRDOSE 3 software and the ability to determine lesion doses to evaluate BG dosimetry. Even though BG structures are not perfect spheres, this approximation may be sufficient for dosimetry purposes. This enabled us to calculate self-dose from BG uptake and determine if injected dosages should be modified to account for these structures. As the maximum dose to BG in one subject exceeded bladder dose by 18%, we are changing our guidelines to limit the maximum permissible injected dosage for ^18^F-FPCIT from 23 mCi to 19 mCi [2].

Even though we use 5 mCi for routine brain imaging with ^18^F-FPCIT, we think that it is not a question of 23 versus 5 mCi, but whether the limit of 23 mCi should be based upon bladder dose or BG dose [2]. We want to stress in this paper that BG should be considered a special structure because dopaminergic tracers bind avidly to these regions and have long residence times. It has been demonstrated that bladder is the dose-limiting organ for these radiotracers which we think is not appropriate. In the future, as we develop new radiotracers with high affinity to BG, especially for SPECT tracers with long half-life nuclides, BG exposure indeed may easily exceed bladder dose and thus become the dose-limiting “organ.” There are no prior studies suggesting that bladder is a very radiosensitive organ but it is still considered dose-limiting. This may be true for BG as well; we just do not have any direct studies on BG radiosensitivity. We have, however, mentioned in the introduction how BG may be more radiosensitive than the rest of the brain. We would prefer to err on the side of caution.

It is likely that F-18 labeled tracers, which take a long time to achieve BG equilibrium as opposed to C-11 labeled tracers, may need special focus on BG dosimetry. For tracers with long half-life, such as I-131 (half-life: 8 days), thyroid dose is high enough to limit the diagnostic (not therapeutic) injection to 4 mCi, which is a suboptimal dose for imaging. Similarly, Mn-52 and Sc-46 (half-lives: 5.6 days and 84 days, resp.), which have been recently considered for nonhuman imaging studies, will have serious limitations as to the injected dose for human diagnostic studies because of long residence times.

In our laboratory, we perform multitracer multiday protocols on the same subject and have to often limit the injected activity well below maximum allowable limit for ^18^F-FPCIT, ^18^F-FDG, and H_2_^15^O in order to remain within radiation exposure guidelines. This compromise forces us to extend the scan time for ^18^F-FDG studies to keep signal-to-noise acceptable.

There are two limitations of the study: (1) no individual MRI estimates of caudate and putamen; and (2) bladder doses for individual subjects not being available. Nonetheless, these two issues are expected to have minor influence on our findings.

From the 16 normal subjects studied, BG appears to be the critical organ for dosimetric consideration in three subjects. In a total of eight subjects, BG dose exceeded 85% of the bladder dose and in three subjects BG dose actually exceeded the bladder dose. As new compounds with high affinity or for similar tracers with long half-life radionuclide label are developed, BG dosimetry should be considered in the development process.

## Figures and Tables

**Table 1:** Radiation absorbed doses to basal ganglia.

Sex	Age	Dose (mGy/MBq)				Average BG dose	BG fractional
		L Cau	R Cau	L Put	R Put	(mGy/MBq)	Dose∗
m	53	0.0444	0.0404	0.0390	0.0406	0.0411	0.6966
f	52	0.0749	0.0489	0.0576	0.0482	0.0574	0.9728
f	23	0.0501	0.0535	0.0431	0.0607	0.0518	0.8785
m	50	0.0304	0.0301	0.0281	0.0283	0.0292	0.4954
f	28	0.0577	0.0559	0.0538	0.0529	0.0551	0.9336
f	23	0.0435	0.0428	0.0410	0.0392	0.0417	0.7060
m	69	0.0688	0.0719	0.0696	0.0670	0.0693	***1.1750***
f	65	0.0359	0.0364	0.0343	0.0345	0.0353	0.5977
f	39	0.0301	0.0734	0.0260	0.0518	0.0453	0.7681
f	56	0.0265	0.0324	0.0345	0.0275	0.0302	0.5125
f	55	0.0479	0.0413	0.0403	0.0381	0.0419	0.7099
f	73	0.0532	0.0423	0.0736	0.0412	0.0526	0.8915
m	47	0.0582	0.0552	0.0556	0.0414	0.0526	0.8914
m	70	0.0479	0.0453	0.0394	0.0380	0.0426	0.7228
f	53	0.0725	0.0725	0.0534	0.0528	0.0628	***1.0648***
f	76	0.0655	0.0657	0.0544	0.0519	0.0594	***1.0063***

Mean		**0.0505**	**0.0505**	**0.0465**	**0.0446**	**0.0480**	**0.8139**
SD		0.0147	0.0138	0.0134	0.0107	0.0112	0.1890

Caudate (Cau); putamen (Put); basal ganglia (BG). ∗Fractional dose is calculated by dividing the BG dose by bladder dose of 0.059 mGy/MBq taken from [2].
